# Comparing exercise modalities during caloric restriction: a systematic review and network meta-analysis on body composition

**DOI:** 10.3389/fnut.2025.1579024

**Published:** 2025-05-29

**Authors:** Yongchao Xie, Yu Gu, Zhen Li, Lei Zhang, Yang Hei

**Affiliations:** ^1^Centre for Sport Nutrition and Health, Centre for Nutritional Ecology, School of Physical Education (Main Campus), Zhengzhou University, Zhengzhou, China; ^2^Henan Sports Medicine and Rehabilitation Center, Henan Sport University, Zhengzhou, China; ^3^Department of Physical Education, College of Education, Seoul National University, Seoul, Republic of Korea

**Keywords:** exercise, caloric restriction, weight, body fat, lean body mass

## Abstract

**Background:**

In recent years, caloric restriction (CR), intermittent fasting (IF), and the ketogenic diet (KD) have gained popularity as primary dietary interventions for improving body composition. While these approaches offer benefits, both IF and KD have been associated with various adverse effects. Although CR is generally devoid of significant side effects, it may lead to reductions in lean body mass. To mitigate this, CR combined with exercise (CR + EX) has become a preferred strategy for maintaining overall health. However, under CR conditions, the effects of exercise may differ from those observed with a normal diet. Most existing studies compare CR + EX with CR alone, exercise alone, or a control (CON) group. Although prior studies have examined caloric restriction with exercise, direct comparisons between specific exercise modalities in a caloric deficit remain unclear, necessitating a network meta-analysis approach. This study summarizes the literature on CR combined with exercise to identify which exercise regimen, when paired with CR, yields the most favorable outcomes. The findings will provide valuable recommendations for individuals seeking to maintain or improve body composition through CR + EX.

**Methods:**

A systematic review was conducted in accordance with the PRISMA guidelines, covering literature from the inception of databases up to September 2024. Searches were performed in PubMed, Web of Science, Embase, and the Cochrane Library. This study was registered in PROSPERO under the identifier: CRD42024588241. Only randomized controlled trials (RCTs) involving healthy populations were included. Articles were rigorously screened according to the PICOS strategy (methods) eligibility criteria, and the risk of bias was assessed using the Cochrane Risk of Bias Tool. A network meta-analysis was performed, and the intervention effects were ranked using the Surface Under the Cumulative Ranking (SUCRA) curve.

**Results:**

The network meta-analysis included 62 RCTs, encompassing 4,429 participants. The ranking of intervention effects is as follows: Weight reduction: high-intensity aerobic exercise (HA) > moderate-intensity aerobic exercise (MA) > low-intensity aerobic exercise (LA) > moderate-intensity mixed exercise (MM) > high-intensity mixed exercise (HM) > CR > low-intensity resistance exercise (LR) > moderate-intensity resistance exercise (MR) > high-intensity resistance exercise (HR) > Control group (CON), Compared to CON, the effect sizes for the other groups were as follows: HA: 7.94 (6.34, 9.55), MA: 7.78 (5.97, 9.58), LA: 7.10 (5.10, 9.10), MM: 6.65 (3.49, 9.81), HM: 7.47 (3.19, 11.75), CR: 7.10 (5.10, 9.10), LR: 5.45 (0.17, 10.72), MR: 5.62 (3.17, 8.06), HR: 6.00 (3.24, 8.76); BMI reduction: LA > MM > HA > MA > HR > CR > HM > MR > CON; Fat mass reduction: LA > HA > HM > MA > MR > LR > HR > CR > MM > CON, Compared to CON, the effect sizes for the other groups were as follows: LA: 2.70 (1.76, 3.65), MM: 3.35 (1.94, 4.75), HA: 2.90 (2.11, 3.70), MA: 2.96 (2.09, 3.84), HR: 2.56 (1.43, 3.69), CR: 2.47 (1.79, 3.15), HM: 1.73 (−0.34, 3.81), MR: 2.26 (1.08, 3.45); Body fat percentage reduction: HA > MM > LR > HR > MR > HM > MA > LA > CR > CON, Compared to CON, the effect sizes for the other groups were as follows: HA: 4.80 (3.50, 6.10), MM: 5.87 (3.22, 8.52), LR: 6.24 (1.05, 11.42), HR: 4.40 (1.93, 6.87), MR: 4.18 (2.21, 6.15), HM: 4.40 (0.80, 7.99), MA: 4.17 (2.70, 5.64), LA: 3.40 (1.44, 5.35), CR: 3.23 (2.08, 4.39); Lean body mass preservation: CON > MM > MR > LR > HR > MA > LA > HM > HA > CR, Compared to CON, the effect sizes of the other groups were as follows: MM: 0.14 (−2.91, 3.19), MR: 0.03 (−2.24, 2.29), LR: 0.36 (−4.15, 4.87), HR: −0.17 (−2.36, 2.02), MA: −0.40 (−2.22, 1.43), LA: −0.58 (−2.75, 1.59), HM: −0.81 (−4.27, 2.65), HA: −0.67 (−2.33, 0.98), CR: −1.66 (−3.12, −0.19). In summary, LR + CR, MA + CR and MR + CR are at an advantageous level in improving various indicators.

**Conclusion:**

Combining moderate-and low-intensity resistance or aerobic exercise with caloric restriction optimizes fat loss while preserving lean body mass, making it a superior strategy for body composition improvement.

**Systematic review registration:**

This study was registered in PROSPERO under the identifier: CRD42024588241.

## 1 Introduction

With the increase in human energy intake, more individuals are struggling with the negative impacts of overweight and obesity. Preventing and addressing obesity has become a key area of growing concern. It is well established that reducing total body weight or body fat by 5–10% can yield significant health benefits. Among non-pharmacological interventions for preventing and managing obesity, diet and exercise are the most widely applicable methods (1–3).

In recent years, caloric restriction (CR), intermittent fasting (IF), and the ketogenic diet (KD) have gained popularity as major dietary interventions. While these approaches can improve body composition, both IF and KD have been associated with various adverse effects. Studies have shown that KD has pro-inflammatory effects, which may lead to heart fibrosis and kidney damage (4–9), often linked to elevated lipid levels in the body (10). Several studies suggest that IF protocols may induce apoptosis through pathways that are independent of caloric reduction, circadian rhythm alterations, or the mTORC1 nutrient-sensing mechanism. Such fasting triggers a rapid surge in free fatty-acid release, disrupts cellular metabolism, elevates reactive oxygen species (ROS), and causes oxidative damage and programmed cell death, ultimately leading to metabolic dysregulation and impaired normal growth. In contrast, studies suggesting adverse side effects of CR are relatively limited. CR has been shown to promote mitochondrial biogenesis, improving mitochondrial function and efficiency, reducing total oxygen consumption, and consequently decreasing ROS production (11). Some studies indicate that CR, when not associated with malnutrition, can extend lifespan in various species (12). Therefore, in an era of rising obesity rates, CR is often considered the most effective method for improving overall health. While CR reduces body fat, many studies suggest it also leads to a decrease in lean body mass (13, 14). Research indicates that diet-induced weight loss reduces both fat mass and fat-free mass (FFM), with approximately 75% of the lost weight being fat tissue and about 25% being FFM (15, 16). Loss of skeletal muscle is particularly concerning, as it may impair exercise capacity, lower basal metabolic rate and calorie expenditure, and is associated with increased disability, hospitalization, and mortality risks (17–19).

Some studies have shown that exercising during CR can reduce the loss of FFM by up to 50% (20). Our previous research confirmed that combining exercise with CR significantly helps preserve lean body mass (21). Other studies have suggested that resistance exercise during CR nearly halts the loss of lean body mass induced by CR (22, 23). Consequently, many studies combine CR with exercise to achieve the dual benefits of reducing body fat while preserving lean body mass. However, during CR, the body’s metabolic state may change, and the effects of different types of exercise may vary. Most studies compare CR + EX with CON, exercise alone, or CR alone, demonstrating significant improvements in body composition, but they fail to compare the effects of different exercise modalities. According to previous literature, We hypothesize that CR + AE will be the most effective for weight and fat loss, while CR + RE will be superior for lean body mass preservation. To categorize the various exercise modalities, we define exercise intensity as follows: Low-intensity exercise was defined as less than 39% of maximal oxygen uptake (VO₂ max), 54% of maximal heart rate (HR max), 39% of heart rate reserve (HRR), and 49% of one-repetition maximum (1RM). Moderate-intensity exercise was defined as falling between the upper limit of low intensity and below 69% of VO₂ max, 74% of HR max, 69% of HRR, and 70% of 1RM. Exercise exceeding the upper limit of moderate intensity was classified as high-intensity exercise (24).

Since factors such as exercise type and intensity can lead to variations in exercise outcomes—and even cause negative effects—it is crucial to explore which exercise modality, when combined with CR, produces the best results. This study summarizes the literature on CR combined with exercise to identify which exercise regimen, when paired with CR, yields the most favorable outcomes. The findings will provide valuable recommendations for individuals seeking to maintain or improve body composition through CR + EX.

## 2 Methods

### 2.1 Protocol and registration

This systematic review was conducted in accordance with the Preferred Reporting Items for Systematic Reviews and Meta-Analyses (PRISMA) guidelines (25). The review protocol has been registered with PROSPERO (identifier: CRD42024588241).

### 2.2 Search strategy

A thorough search was conducted from the inception of the databases to September 2024, covering PubMed, Web of Science, Embase, and the Cochrane Library. Only English-language publications were considered, this network meta-analysis did not include gray literature or unpublished studies. Two authors (Y.X. and Y.H.) independently reviewed and assessed relevant articles according to predefined inclusion criteria. Disagreements regarding article selection were resolved through discussion with a third author (L.Z.). The search terms used are listed in Supplementary file S1.

### 2.3 Inclusion criteria and exclusion criteria

Inclusion Criteria: (1) the included literature must be a randomized controlled trial containing at least two groups, one of which must be a CR + EX group, and the other groups can be a CON group, a CR group, or a CE + EX group. (2) Participants must be healthy individuals. (3) Outcome measures must include at least one of the following: Weight, BMI, body fat percentage, body fat mass or lean body mass.

Exclusion Criteria: (1) studies that are not randomized controlled trials. (2) Non-human studies. (3) Non-original studies, such as reviews, letters, case reports, or papers lacking accurate and clear data. (4) Studies involving individuals with existing diseases.

### 2.4 Data extraction

Data extraction from the selected studies was performed using a pre-structured Excel 2019 spreadsheet. The extracted information included study titles, participant demographics, age, intervention methods, duration, and quality assessments. Two authors (Y.H. and Y.X.) independently carried out the data extraction, with discrepancies resolved through consultation with a third author (Y.G.). For studies that presented mean values solely in graphical form, the Web Plot Digitizer (2024.09)^1^ was used to extract the data. When data were not presented as “Mean ± SD,” a standardized evidence-based medicine template was applied to convert them into the appropriate format.

### 2.5 Risk of bias assessment

The quality of the included RCTs was assessed using the Cochrane Risk of Bias Tool, which evaluates seven domains: random sequence generation and allocation concealment (selection bias), blinding of participants and personnel (performance bias), blinding of outcome assessment (detection bias), incomplete outcome data (attrition bias), selective reporting (reporting bias), and other biases. Each domain was classified as having low, high, or unclear risk of bias. Two authors (Y.X. and Y.H.) independently conducted the assessments, with any disagreements resolved through discussion with a third author (L.Z.).

### 2.6 Statistical analysis

Review Manager 5.3 was used to analyze the data and generate both the Risk of Bias summary and the Risk of Bias graph. Network meta-analysis combines direct and indirect evidence, thereby increasing statistical power and alleviating problems caused by sparse data. The surface under the cumulative ranking curve (SUCRA) quantifies the probability distribution of treatment ranks and is particularly valuable for its clinical interpretability. By converting cumulative ranking probabilities into a 0–100% scale, SUCRA conveys the overall hierarchy of therapeutic benefit more clearly than simply reporting the “best-rank” probability. In addition, because it is based on cumulative probabilities, SUCRA is less sensitive to marginal differences between ranks and thus helps prevent over-interpretation of trivial distinctions. Network meta-analysis, consistency testing between direct and indirect comparisons, and the calculation of the League Table for interventions were performed using Stata MP 17 software. Surface Under the Cumulative Ranking (SUCRA) values were also calculated, and various plots, including the Network Plot, SUCRA Plot, clustered ranking plot, and funnel plots, were generated. Results were presented as standardized mean differences (SMD) with 95% confidence intervals (95% CI). We will assess the data for inconsistency; when significant inconsistency is detected, we will first perform subgroup analyses. Sensitivity analyses will be undertaken only if the subgroup approach fails to resolve the discrepancies.

## 3 Results

### 3.1 Search results and study selection

**Figure 1:**
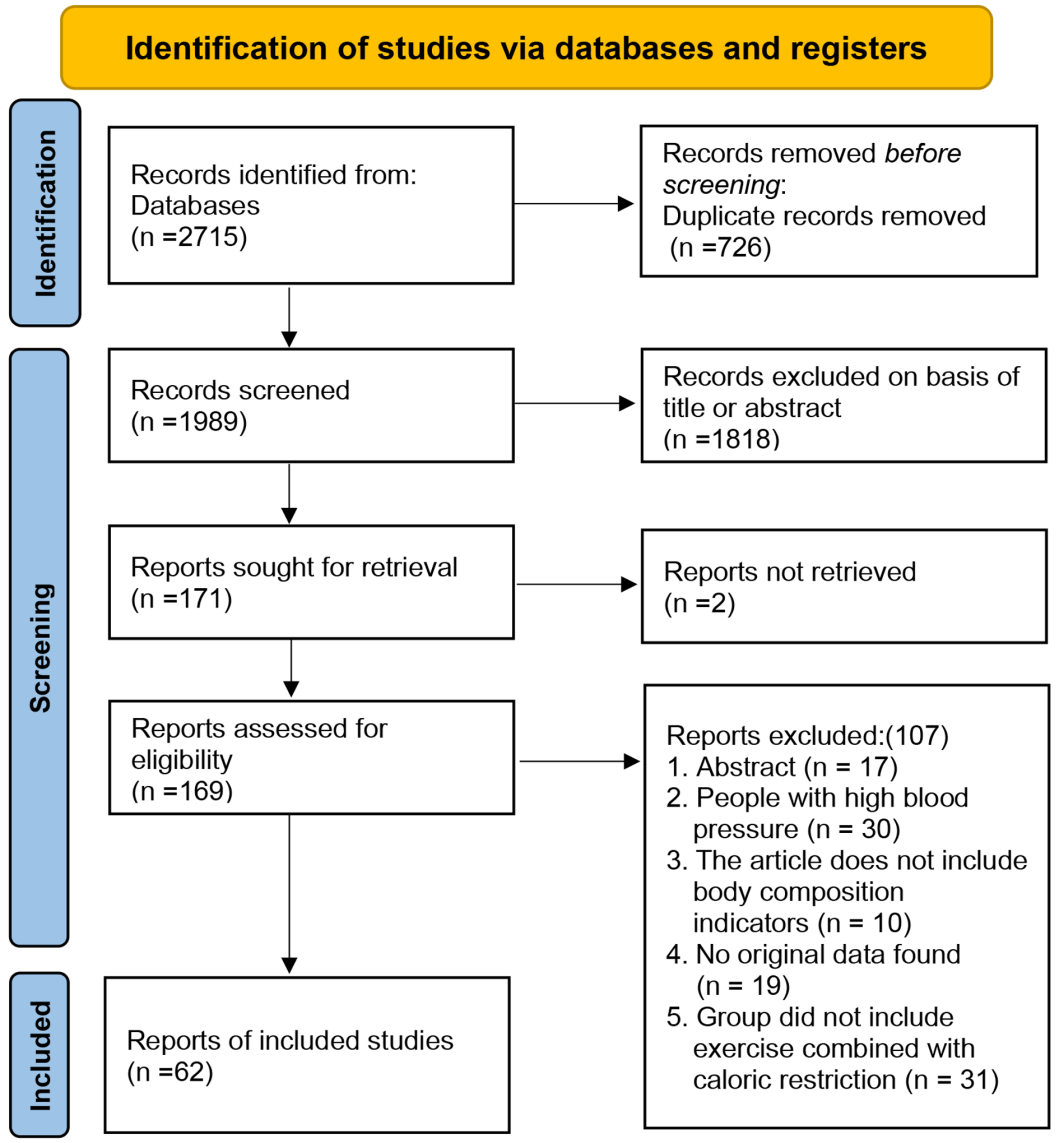
Flow diagram of study selection.

As of September 2024, a total of 2,715 articles were retrieved from four databases: PubMed (240), Web of Science (1,951), Embase (272), and the Cochrane Library (252). After removing 726 duplicates, 1,989 articles were excluded based on their titles or abstracts, leaving 171 articles for full-text review. However, 2 articles could not be located prior to the full-text review. Following the review, 169 articles were excluded for various reasons: abstract-only content (*n* = 17), participants with high blood pressure (*n* = 30), lack of body composition indicators (*n* = 10), absence of original data (*n* = 19), and studies not involving exercise combined with dietary interventions (*n* = 31). In total, 62 articles were included in the final analysis (Figure 1) (11, 17, 26–85). The screening process was independently conducted by two authors, resulting in a kappa value of 0.7618, indicating a high level of agreement.

### 3.2 Study characteristics

A total of 62 articles were included in the meta-analysis (Supplementary Table S1). These studies, conducted between 1995 and 2024, involved 4,429 participants. The duration of exercise interventions ranged from 2 weeks to 1 year, with the primary exercise modalities being resistance training and aerobic activities, including running, cycling, walking, and swimming. Exercise intensity was classified into low, moderate, and high intensity. The exercise modes examined in this study include low-intensity aerobic exercise (LA), low-intensity resistance exercise (LR), moderate-intensity aerobic exercise (MA), moderate-intensity resistance exercise (MR), moderate-intensity mixed exercise (MM), high-intensity aerobic exercise (HA), high-intensity resistance exercise (HR), and high-intensity mixed exercise (HM). Each exercise session lasted between 30 and 60 min, with participants exercising 2 to 7 times per week. The studies reviewed reported potential side effects during the initial phase of CR, such as irritability, difficulty concentrating, and reduced energy levels. However, these symptoms typically improved as participants’ bodies adapted to the intervention. In the CR group, total energy expenditure (TEE) was measured 1 week before the intervention began. During the intervention, participants in the CR group had their caloric intake reduced by 10–25% of their TEE. All dietary interventions were designed by professional nutritionists. In most studies, participants were provided with 1–2 main meals per day or were instructed to record the types and quantities of food they consumed daily. The specific characteristics of the studies are detailed in Supplementary Table S1.

### 3.3 Risk of bias in included studies

Among the 62 included studies, 3 could not be assessed for the risk of random sequence generation, 39 could not be assessed for allocation concealment, and 8 could not be evaluated for incomplete outcome data. All studies that were assessed were deemed to have an unclear risk for blinding of participants and personnel, as effective blinding of the dietary and exercise interventions in the experimental groups was unlikely (Supplementary Figures S1, S2).

### 3.4 Effects of the interventions

We first conducted a consistency test across multiple interventions. When significant inconsistency was identified, subgroup analyses were performed to investigate potential sources, focusing on factors such as gender, age, obesity, intervention duration, and frequency. If consistency was deemed adequate, a Network Plot was generated to visualize the distribution of key characteristics across the studies. Subsequently, a League Table was created to present pairwise comparison data, followed by the construction of a SUCRA Plot to rank the different intervention methods. Even when the study population and exercise modality are identical, a certain degree of heterogeneity may still emerge in the outcomes. For instance, variability in high-intensity resistance-training results can stem from differences in participants’ baseline training status, adherence levels, or methodological inconsistencies across studies.

#### 3.4.1 The effect of different exercise combined with caloric restriction on body weight

**Figure 2:**
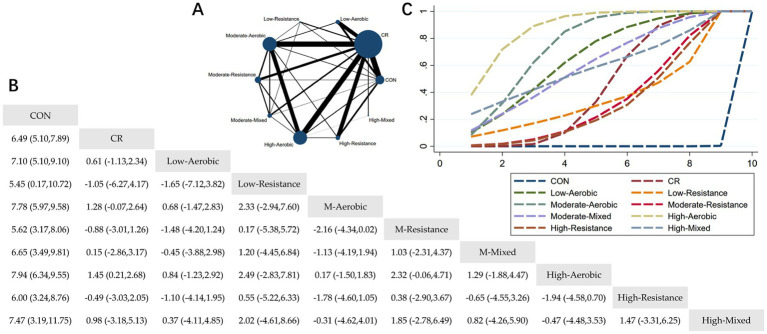
Network meta-analysis of weight: Network Plot, League Table, and SUCRA Plot. **(A)** Presents the Network Plot. The size of the nodes is proportional to the sample size of each dietary intervention, and the thickness of the lines corresponds to the number of available studies. **(B)** Displays the pairwise comparison League Table, where the estimated effect size differences (SMD with 95% CI) represent the difference between the intervention on the top and the intervention on the right. **(C)** Illustrates the SUCRA Plot, where the size of the area under the curve indicates the effectiveness of each intervention.

The Network Plot, League Table, and SUCRA Plot for the effect of different exercise combined with caloric restriction on body weight are shown in Figure 2.

As shown in Figure 2A, most studies primarily investigated caloric restriction combined with low-intensity aerobic exercise, moderate-intensity aerobic exercise, high-intensity aerobic exercise, and high-intensity resistance exercise. The largest number of participants was found in the caloric restriction combined with moderate-intensity aerobic exercise and caloric restriction combined with high-intensity aerobic exercise groups. Figure 2B summarizes the estimated effect size differences (SMD, 95% CI) for pairwise comparisons of 10 intervention methods. Compared to the control group, all 9 intervention groups showed significant reductions in body weight. When compared to the caloric restriction group, only the caloric restriction combined with high-intensity aerobic exercise group showed a significant reduction in body weight. Figure 2C displays the SUCRA ranking of the 10 interventions, with the area under the curve representing the effectiveness of each intervention. The larger the area under the curve, the higher the effectiveness ranking. The ranking of interventions for weight loss was as follows: HA > MA > LA > MM > HM > CR > LR > MR > HR > CON. Compared to CON, the effect sizes for the other groups were as follows: HA: 7.94 (6.34, 9.55), MA: 7.78 (5.97, 9.58), LA: 7.10 (5.10, 9.10), MM: 6.65 (3.49, 9.81), HM: 7.47 (3.19, 11.75), CR: 7.10 (5.10, 9.10), LR: 5.45 (0.17, 10.72), MR: 5.62 (3.17, 8.06), HR: 6.00 (3.24, 8.76).

#### 3.4.2 The effect of different exercise combined with caloric restriction on BMI

**Figure 3:**
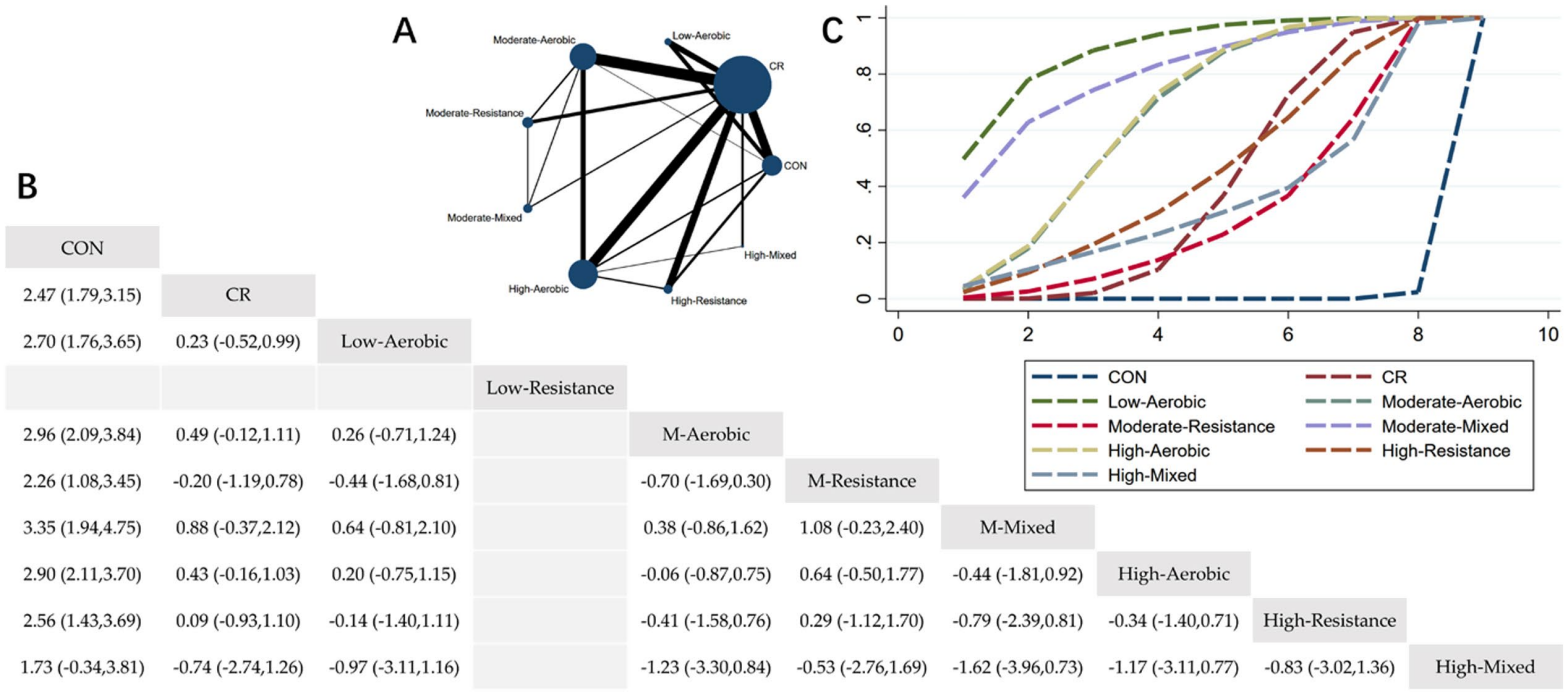
Network meta-analysis of BMI: Network Plot, League Table, and SUCRA Plot. **(A)** Presents the Network Plot. The size of the nodes is proportional to the sample size of each intervention, and the thickness of the lines corresponds to the number of available studies. **(B)** Displays the pairwise comparison League Table, where the estimated effect size differences (SMD with 95% CI) represent the difference between the intervention on the top and the intervention on the right. **(C)** Illustrates the SUCRA Plot, where the size of the area under the curve indicates the effectiveness of each intervention.

The Network Plot, League Table, and SUCRA Plot for the effect of different exercise combined with caloric restriction on BMI are shown in Figure 3.

As shown in Figure 3A, most studies primarily investigated caloric restriction combined with low-intensity aerobic exercise, moderate-intensity aerobic exercise, high-intensity aerobic exercise, and high-intensity resistance exercise. The largest number of participants were in the caloric restriction combined with moderate-intensity aerobic exercise and caloric restriction combined with high-intensity aerobic exercise groups. Figure 3B summarizes the estimated effect size differences (SMD, 95% CI) for pairwise comparisons of 9 intervention methods. Compared to the control group, all groups—except for the HM + CR group—showed a significant reduction in BMI. Figure 3C displays the SUCRA ranking of the 9 interventions, with the area under the curve representing the effectiveness of each intervention. The larger the area under the curve, the higher the effectiveness ranking. The ranking of interventions for reducing BMI was as follows: LA > MM > HA > MA > HR > CR > HM > MR > CON. Compared to CON, the effect sizes for the other groups were as follows: LA: 2.70 (1.76, 3.65), MM: 3.35 (1.94, 4.75), HA: 2.90 (2.11, 3.70), MA: 2.96 (2.09, 3.84), HR: 2.56 (1.43, 3.69), CR: 2.47 (1.79, 3.15), HM: 1.73 (−0.34, 3.81), MR: 2.26 (1.08, 3.45).

#### 3.4.3 The effect of different exercise combined with caloric restriction on body fat

**Figure 4:**
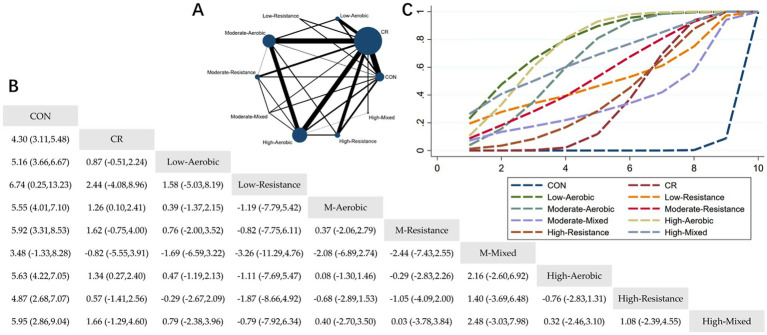
Network meta-analysis of body fat: Network Plot, League Table, and SUCRA Plot. **(A)** Presents the Network Plot. The size of the nodes is proportional to the sample size of each intervention, and the thickness of the lines corresponds to the number of available studies. **(B)** Displays the pairwise comparison League Table, where the estimated effect size differences (SMD with 95% CI) represent the difference between the intervention on the top and the intervention on the right. **(C)** Illustrates the SUCRA Plot, where the size of the area under the curve indicates the effectiveness of each intervention.

The Network Plot, League Table, and SUCRA Plot for the effect of different exercise combined with caloric restriction on body fat are shown in Figure 4.

As shown in Figure 4A, most of the studies primarily investigated caloric restriction combined with low-intensity aerobic exercise, moderate-intensity aerobic exercise, high-intensity aerobic exercise, and high-intensity resistance exercise. The highest number of participants were in the caloric restriction combined with moderate-intensity aerobic exercise and caloric restriction combined with high-intensity aerobic exercise groups. Figure 4B summarizes the estimated effect size differences (SMD, 95% CI) for pairwise comparisons of 10 intervention methods. Compared to the control group, all 8 intervention groups, except for the MM + CR group, showed significant reductions in body fat. Compared to the CR group, the MA + CR and HA + CR groups also showed significant reductions in body fat. Figure 4C displays the SUCRA ranking of the 10 interventions, with the area under the curve representing the effectiveness of each intervention. The larger the area under the curve, the higher the effectiveness ranking. The ranking of interventions for reducing body fat was as follows: LA > HA > HM > MA > MR > LR > HR > CR > MM > CON. Compared to CON, the effect sizes for the other groups were as follows: LA: 5.16 (3.66, 6.67), HA: 5.63 (4.22, 7.05), HM: 5.95 (2.86, 9.04), MA: 5.55 (4.01, 7.10), MR: 5.92 (3.31, 8.53), LR: 6.74 (0.25, 13.23), HR: 4.87 (2.68, 7.07), CR: 4.30 (3.11, 5.48), MM: 3.48 (−1.33, 8.28).

#### 3.4.4 The effect of different exercise combined with caloric restriction on body fat percentage

**Figure 5:**
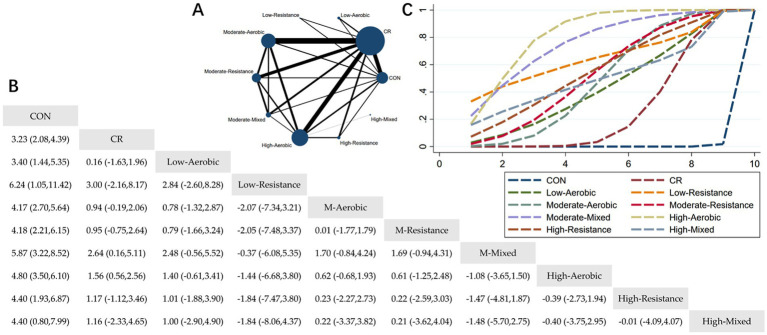
Network meta-analysis of body fat percentage: Network Plot, League Table, and SUCRA Plot. **(A)** Presents the Network Plot. The size of the nodes is proportional to the sample size of each intervention, and the thickness of the lines corresponds to the number of available studies. **(B)** Displays the pairwise comparison League Table, where the estimated effect size differences (SMD with 95% CI) represent the difference between the intervention on the top and the intervention on the right. **(C)** Illustrates the SUCRA Plot, where the size of the area under the curve indicates the effectiveness of each intervention.

The Network Plot, League Table, and SUCRA Plot for the effect of different exercise combined with caloric restriction on body fat percentage are shown in Figure 5.

As shown in Figure 5A, most studies primarily investigated caloric restriction combined with moderate-intensity aerobic exercise, moderate-intensity resistance exercise, and high-intensity aerobic exercise. The largest number of participants were in the MA + CR, MR + CR, and HA + CR groups. Figure 5B summarizes the estimated effect size differences (SMD, 95% CI) for pairwise comparisons of 10 intervention methods. Compared to the control group, all 9 intervention groups showed significant reductions in body fat percentage. Compared to the CR group, the MM + CR and HA + CR groups demonstrated significant reductions in body fat percentage. Figure 5C displays the SUCRA ranking of the 10 interventions, with the area under the curve representing the effectiveness of each intervention. The larger the area under the curve, the higher the effectiveness ranking. The ranking of interventions for reducing body fat percentage was as follows: HA > MM > LR > HR > MR > HM > MA > LA > CR > CON. Compared to CON, the effect sizes for the other groups were as follows: HA: 4.80 (3.50, 6.10), MM: 5.87 (3.22, 8.52), LR: 6.24 (1.05, 11.42), HR: 4.40 (1.93, 6.87), MR: 4.18 (2.21, 6.15), HM: 4.40 (0.80, 7.99), MA: 4.17 (2.70, 5.64), LA: 3.40 (1.44, 5.35), CR: 3.23 (2.08, 4.39).

#### 3.4.5 The effect of different exercise combined with caloric restriction on lean mass

**Figure 6:**
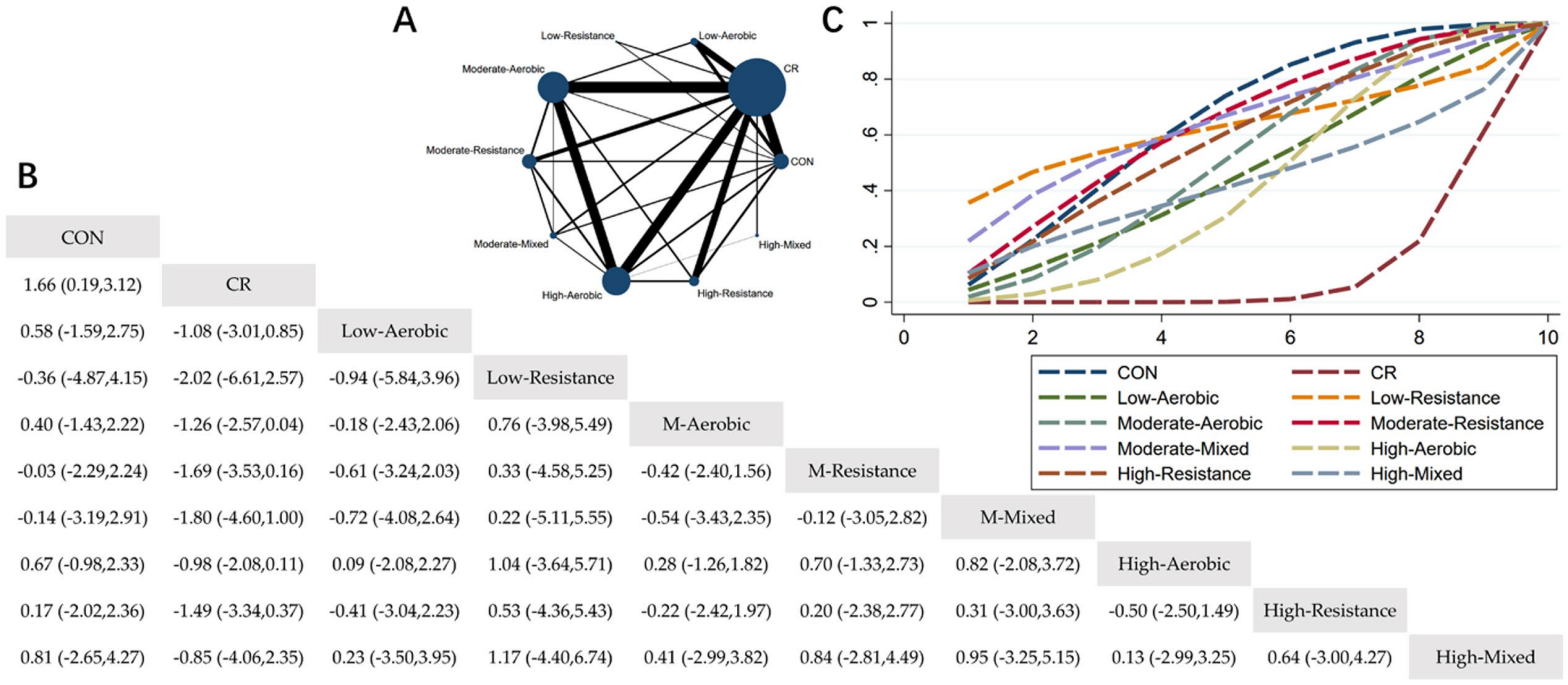
Network meta-analysis of lean mass: Network Plot, League Table, and SUCRA Plot. **(A)** Presents the Network Plot. The size of the nodes is proportional to the sample size of each intervention, and the thickness of the lines corresponds to the number of available studies. **(B)** Displays the pairwise comparison League Table, where the estimated effect size differences (SMD with 95% CI) represent the difference between the intervention on the top and the intervention on the right. **(C)** Illustrates the SUCRA Plot, where the size of the area under the curve indicates the effectiveness of each intervention.

The Network Plot, League Table, and SUCRA Plot for the effect of different exercise combined with caloric restriction on lean mass are shown in Figure 6.

As shown in Figure 6A, most of the studies primarily focused on caloric restriction combined with low-intensity aerobic exercise, moderate-intensity aerobic exercise, high-intensity aerobic exercise, and high-intensity resistance exercise. The MA + CR and HA + CR groups had the largest number of participants. Figure 6B summarizes the estimated effect size differences (SMD, 95% CI) from pairwise comparisons of the 10 intervention methods. Compared to the control group, the CR group showed a significant decrease in lean body mass. Additionally, the MM + CR and HA + CR groups demonstrated a significant decrease in lean body mass compared to the CR group. Figure 6C presents the SUCRA rankings of the 10 interventions, where the area under the curve represents the effectiveness of each intervention. The larger the area under the curve, the higher the effectiveness ranking. The ranking of interventions in maintaining lean body mass was as follows: CON > MM > MR > LR > HR > MA > LA > HM > HA > CR. Compared to CON, the effect sizes of the other groups were as follows: MM: 0.14 (−2.91, 3.19), MR: 0.03 (−2.24, 2.29), LR: 0.36 (−4.15, 4.87), HR: −0.17 (−2.36, 2.02), MA: −0.40 (−2.22, 1.43), LA: −0.58 (−2.75, 1.59), HM: −0.81 (−4.27, 2.65), HA: −0.67 (−2.33, 0.98), CR: −1.66 (−3.12, −0.19).

#### 3.4.6 The effect of different interventions on LM + FAT

**Figure 7:**
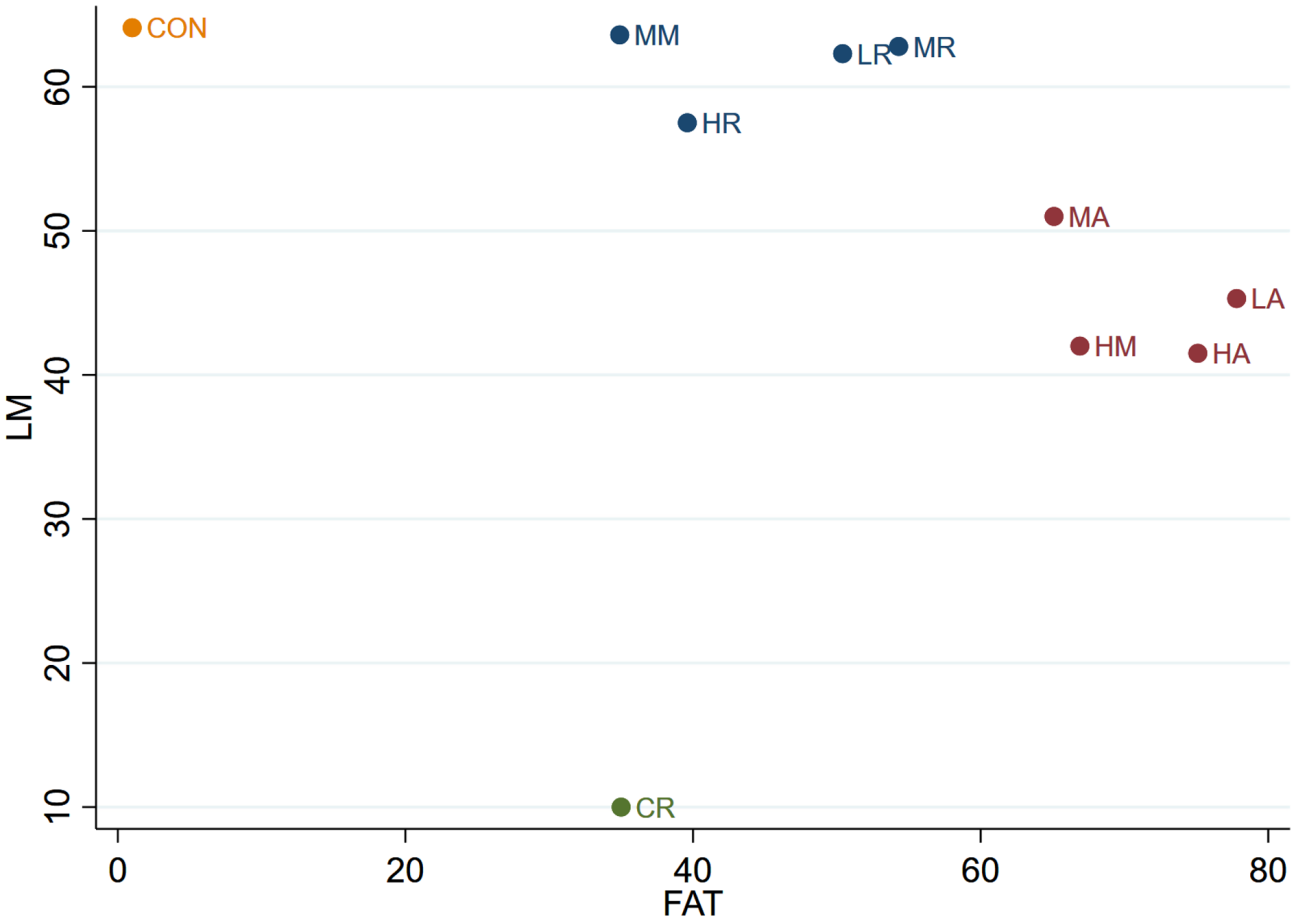
The Clustered Ranking Plot of LM + FAT. The horizontal axis represents the effect on reducing FAT, and the vertical axis represents the effect on increasing LM, with points in the top right representing the best effects.

The Clustered Ranking Plot for the effects of different interventions on LM + FAT is shown in Figure 7.

As shown in Figure 7, the 10 interventions are categorized into four groups. The CON group is the most effective in maintaining lean body mass but the least effective in reducing body fat. The CR group exhibits moderate effectiveness in reducing body fat but is the least effective in preserving lean body mass. The LR, MR, HR, and MM groups form a category that is effective in preserving lean body mass but only moderately effective in reducing body fat. The LA, MA, HA, and HM groups are grouped together in another category, which is effective in reducing body fat but only moderately effective in maintaining lean body mass.

## 4 Discussion

Our network meta-analysis (NMA) included 62 randomized controlled trials to evaluate the effects of CR and CR combined with exercise on body composition. Based on the included studies, we ranked the effects of 10 interventions (CON, CR, LA + CR, LR + CR, MA + CR, MR + CR, MM + CR, HA + CR, HR + CR, and HM + CR) to assess the differences in their impacts on body composition indicators. Our findings suggest that for weight reduction, aerobic exercise is the most effective, with its efficacy increasing with exercise intensity. In contrast, resistance training is less effective for weight reduction compared to CR alone. For body fat reduction, aerobic exercise is more effective than resistance training and combined exercise regimens. In terms of preserving lean body mass, the effects under CR differed from those observed with a normal diet. During CR, moderate-intensity mixed exercise and all intensities of resistance training were the most effective at maintaining lean body mass. However, as the intensity of mixed exercise and aerobic exercise increased, their effects on lean body mass became less favorable. Each intervention effectively reduced body fat and weight but also resulted in a reduction in lean body mass. Overall, LR + CR, MA + CR, and MR + CR demonstrated the most favorable combined effects in terms of reducing body fat and preserving lean body mass.

It is well established that exercise improves physical fitness; however, the effects of exercise during CR are less understood. CR induces a negative energy balance by reducing caloric intake, which results in changes to energy metabolis (86–88). When energy intake persistently falls below energy expenditure, the body enters a state of negative energy balance. To bridge the resulting caloric gap, it not only mobilizes adipose reserves but also catabolizes skeletal muscle proteins via gluconeogenesis to produce glucose, thereby eroding muscle mass. The concomitant reduction in lean tissue is accompanied by a decline in resting metabolic rate. Collectively, these metabolic adaptations shift the organism toward a survival-oriented physiological profile. This metabolic adaptation seems to be unique to CR, as studies on exercise-induced weight loss have not observed a similar adaptation (89–92). The focus of this study is to explore the effects of exercise during this period of metabolic change.

Our findings suggest that, during CR, aerobic exercise is more effective than combined exercise or resistance training for weight reduction. Both aerobic and resistance exercises contribute to reductions in body fat. However, aerobic exercise demands more energy, and during CR, this additional energy is sourced from fat stores, making it more effective than resistance training for reducing overall body weight. Additionally, resistance training tends to increase muscle mass, leading to less weight loss compared to aerobic exercise. Some studies propose that, when energy expenditure from exercise is equal, the intensity of exercise may not significantly influence weight loss (71). However, our results indicate that weight loss improves as the intensity of aerobic exercise increases. This is not contradictory, as the studies included in this analysis did not directly measure energy expenditure during exercise. Exercise durations ranged from 30 to 60 min, with no significant differences in the time spent exercising at different intensities. Consequently, higher intensity resulted in greater energy expenditure. This does not imply that higher intensity is always superior, as intensity was adjusted according to participants’ fitness levels, with no exercise intensity exceeding what participants could safely handle. When exercise intensity surpasses an individual’s capacity, it may lead to adverse effects such as oxidative stress and inflammation, which can negatively impact health (93). This study also suggests that resistance exercise intensity does not significantly affect weight loss during CR. This may be due to the balance between muscle gain and fat loss induced by varying resistance training intensities, which results in no significant change in body weight (94). This finding aligns with some existing literature, which indicates that resistance exercise alone may even lead to weight gain (95). Regarding BMI, the results of this study indicate that changes in BMI follow a similar pattern to changes in body weight.

Adipose tissue serves as the primary energy storage site and is one of the main tissues affected during periods of negative energy balance (96–99). Weight loss resulting from a low-calorie diet differs significantly from weight loss due to exercise. Studies show that a 5% reduction in body weight is associated with a 21.3% reduction in body fat following exercise training, compared to only a 13.4% reduction with a low-calorie diet. To achieve the same 13.4% reduction in body fat through exercise, only a 2.4% weight reduction is required. This finding aligns with the ACSM position statement on weight loss interventions, which emphasizes the broader health benefits of exercise beyond weight reduction (100). Clinically significant reductions in body fat (up to 6.1%) can occur without any weight loss following exercise training, potentially reducing cardiovascular risk and improving metabolic health (101). However, some studies suggest that long-term caloric restriction can also produce fat loss comparable to exercise (102). Nonetheless, combined interventions of caloric restriction and exercise are generally more effective in reducing body fat. Our study confirms that, under caloric restriction, aerobic exercise is more effective in reducing body fat than combined exercise or resistance training, aligning with trends observed under normal dietary conditions. Once aerobic exercise reaches a sufficient duration, the body predominantly relies on fat as an energy source, making aerobic exercise the most effective for fat reduction. In contrast, resistance training, though less energy-intensive, promotes muscle growth, which increases TEE and indirectly enhances fat utilization (103, 104). Several studies suggest that improvements in lipid metabolism are linked to the volume of physical activity, emphasizing the importance of energy expenditure in enhancing lipid metabolism (105, 106), which supports our findings. Interestingly, our results show that moderate-to low-intensity resistance training is more effective than high-intensity resistance training in reducing body fat during caloric restriction. From an energy expenditure perspective, resistance training intensity has minimal impact on energy consumption, as lifting a weight equal to 1RM once requires the same energy as lifting 50% of 1RM for multiple repetitions. Low-intensity resistance training typically involves more repetitions than high-intensity training, resulting in similar energy expenditure between the two. Despite this, we observed that the CR + HR combination was less effective in maintaining lean body mass compared to moderate-to low-intensity resistance training. This further reinforces the idea that resistance exercise increases basal metabolic rate by preserving lean body mass, ultimately enhancing fat reduction.

Studies indicate that during CR, the negative energy balance caused by reduced caloric intake leads to decreased mTOR signaling (107). mTORC1 regulates key anabolic processes, including ribosome biogenesis, protein translation, autophagy, lipogenesis, and nucleotide biosynthesis (108). Reduced mTOR activity impairs muscle protein synthesis and promotes protein degradation, leading to a loss of lean body mass (109). When exercise is performed during CR, the body not only enhances fat consumption but also uses energy to support myofibrillar protein synthesis, helping preserve lean body mass (110). Some studies suggest that CR increases PGC-1 and mitochondrial biogenesis, which can improve skeletal muscle mass and function, particularly during short-term CR (111–116). Our study found that during CR, all intensities of resistance training and moderate-intensity combined exercise are most effective in maintaining lean body mass. This is supported by previous research showing that combining resistance and aerobic exercise during CR improves skeletal muscle strength and mass (38). Interestingly, we observed that Moderate-and low-intensity resistance training during caloric restriction effectively preserves lean body mass, outperforming high-intensity resistance training. Resistance training intensity is based on the participant’s 1RM percentage, so there is no concern about excessively high resistance training intensity. Under normal dietary conditions, muscle mass increases with higher resistance training intensity (94, 117). However, during CR, limited energy intake may hinder muscle synthesis, especially with high-intensity resistance training. This phenomenon may stem from an energy deficit that precipitates a latent negative nitrogen balance. Additionally, we found that high-intensity combined exercise and high-intensity aerobic exercise were less effective than other exercise modalities in preserving lean body mass during CR. This contrasts with patterns observed under normal dietary conditions, where higher exercise intensity typically leads to better preservation of lean body mass (118). During CR, high-intensity aerobic exercise may promote protein utilization or even contribute to muscle breakdown, a topic that warrants further investigation. Overall, our findings suggest that the optimal exercise intensity for maintaining lean body mass during CR is lower than typically seen under normal dietary conditions. This aligns with previous studies indicating that excessive resistance training intensity does not necessarily result in better muscle gain than moderate- or low-intensity resistance training (119–121). Individuals performing RT to build LM should avoid prolonged energy deficiency, and individuals performing RT to preserve LM during weight loss should avoid energy deficits >500 kcal day-1 (122), further supporting the conclusions of our study.

Body fat percentage is primarily influenced by fat mass and lean body mass. The results of this study indicate that, under CR, HA is most effective in reducing body fat percentage. This effect may be attributed to the combined impact of CR and HA, which is highly effective in reducing both body weight and fat mass, though less so in preserving lean body mass. During caloric restriction, the body enters a catabolic state, where it breaks down both fat and muscle tissue for energy. However, during high-intensity aerobic exercise, the body preferentially oxidizes fat for fuel. If the caloric deficit is too large or the diet is unbalanced, muscle tissue can also be broken down. While lean body mass decreases, fat mass decreases more significantly, leading to a net reduction in body fat percentage. Furthermore, our results show that, under caloric restriction, resistance exercise is more effective in reducing body fat percentage than moderate-to low-intensity aerobic exercise. This is likely because resistance exercise helps preserve lean body mass, which contributes to maintaining body weight and ultimately results in a lower body fat percentage compared to moderate-to low-intensity aerobic exercise.

In summary, combined interventions such as LR + CR, MA + CR, and MR + CR are the most effective for reducing body fat and maintaining lean body mass. While fat loss is a key goal, preserving lean body mass is equally crucial. Therefore, CR combined with resistance exercise is a superior approach, as it minimizes the loss of lean body mass (95). Some studies suggest that long-term CR can result in fat loss comparable to exercise, but it often leads to significant lean body mass loss (123). Thus, during CR, the emphasis should be on preserving lean body mass. For body recomposition, several studies emphasize that the primary research focus is to identify intervention strategies capable of simultaneously reducing body fat while preserving or increasing lean mass (124–126). The results of this study indicate that moderate-to low-intensity resistance exercise is highly effective in minimizing lean body mass loss while simultaneously reducing body fat. This makes it one of the most effective strategies for improving body composition. In addition, moderate-intensity aerobic exercise also yields positive results by reducing body fat while preserving some lean body mass, though it is less effective than resistance exercise.

These findings indicate that non pharmacological interventions in healthy individuals should be tailored to participant characteristics and the planned duration of treatment. For reducing body weight and body fat percentage, HA + CR is the preferred strategy, whereas MM + CR is better suited for maintaining or augmenting lean body mass. Clinicians should tailor exercise prescriptions based on patient goals.

While many studies show that combined exercise regimens are more effective than either aerobic or resistance exercise alone, this study focused exclusively on body composition outcomes and did not address cardiovascular health. This limitation restricts the broader applicability of our findings. In the studies reviewed, research on resistance and mixed exercise was significantly less prevalent than that on aerobic exercise, which may introduce limitations when integrating the findings across studies. After bouts of high-intensity exercise, the body continues to expend additional energy, leading to an elevated metabolic rate for several hours post-workout—a residual effect that is difficult to eliminate in most experimental settings. Additional factors may also compromise the precision of the findings, including small sample sizes in some RCTs, heterogeneity in caloric-restriction protocols across studies, and a paucity of long-term follow-up data. For future research, it is advisable to investigate the long-term effects of different training modalities and to assess hormonal and metabolic responses to CR + EX interventions.

## 5 Conclusion

Combining moderate-and low-intensity resistance or aerobic exercise with caloric restriction optimizes fat loss while preserving lean body mass, making it a superior strategy for body composition improvement. Suggest future RCTs with standardized exercise protocols to validate findings.

## Data Availability

The datasets presented in this study can be found in online repositories. The names of the repository/repositories and accession number(s) can be found in the article/Supplementary material.

## Footnotes

^1^ https://automeris.io/
