# Supplementary material for: Comparing exercise modalities during caloric restriction: a systematic review and network meta-analysis on body composition
Source: Front Nutr. 2025 May 29;12:1579024. doi: 10.3389/fnut.2025.1579024 (PMC12158682; doi:10.3389/fnut.2025.1579024)
Supplement: Supplementary file 1 [file Supplementary_file_1.docx]

Supplementary Material

# Supplementary File S1. Searching Strategy

**PubMed**

("Exercise"[Mesh]) AND ("Caloric Restriction"[Mesh]) AND (("Body Weight"[Mesh]) OR ("Body Mass Index"[Mesh]) OR (Body Fat) OR (Body Fat Percentage) OR (Lean Body Mass)) AND ((Randomized Controlled Trial [Publication Type]) OR (Randomized [Title/Abstract]) OR (Placebo [Title/Abstract] OR (RCT [Title/Abstract]))

**Web of Science**

#1

TS=(Exercise)OR TS=(Exercises)OR TS=(Physical Activity)OR TS=(Activities, Physical)OR TS=(Activity, Physical)OR TS=(Physical Activities)OR TS=(Exercise, Physical)OR TS=(Exercises, Physical)OR TS=(Physical Exercise)OR TS=(Physical Exercises)OR TS=(Acute Exercise)OR TS=(Acute Exercises)OR TS=(Exercise, Acute)OR TS=(Exercises, Acute)OR TS=(Exercise, Isometric)OR TS=(Exercises, Isometric)OR TS=(Isometric Exercises)OR TS=(Isometric Exercise)OR TS=(Exercise, Aerobic)OR TS=(Aerobic Exercise)OR TS=(Aerobic Exercises)OR TS=(Exercises, Aerobic)OR TS=(Exercise Training)OR TS=(Exercise Trainings)OR TS=(Training, Exercise)OR TS=(Trainings, Exercise)

#2

TS= (Caloric Restriction) OR TS= (Restriction, Caloric) OR TS= (Calorie Restricted Diet) OR TS= (Calorie Restricted Diets) OR TS= (Diet, Calorie Restricted) OR TS= (Restricted Diet, Calorie) OR TS= (Caloric Restricted) OR TS= (Restricted, Caloric) OR TS= (Low-Calorie Diet) OR TS= (Diet, Low-Calorie) OR TS= (Low Calorie Diet) OR TS= (Low-Calorie Diets)

#3

TS= (Body Weight) OR TS= (Body Weights) OR TS= (Weight, Body) OR TS= (Weights, Body)

#4

TS= (Body Mass Index) OR TS= (Index, Body Mass) OR TS= (Quetelet Index) OR TS= (Index, Quetelet) OR TS= (Quetelet's Index) OR TS= (Quetelets Index)

#5

TS= (Body Fat) OR TS= (Fat) OR TS= (Fat Mass) OR TS= (Visceral Fat) OR TS= (Total Body Fat) OR TS= (Adipose Tissue) OR TS= (Subcutaneous Fat)

#6

TS= (Body Fat Percentage)

#7

TS= (Fat-Free Mass) OR TS= (Muscle Mass) OR TS= (Non-Fat Mass) OR TS= (Free-Fat Mass)

#8

TS= (Randomized Controlled Trial) OR TS= (Randomized) OR TS= (Placebo)

#9

#3 OR #4 OR #5 OR #6 OR #7

#10

#1 OR #2 OR #8 OR #8 OR #9

**Embase**

#1

'Exercise'/exp

#2

'Caloric Restriction'/exp

#3

'Body Weight'/exp OR 'Body Mass Index'/exp OR 'Body Fat'/exp OR 'Body Fat Percentage'/exp OR 'Free Fat Mass'/exp

#4

'Randomized Controlled Trial'/exp

#5

#1 AND #2 AND #3 AND #4

**Cochrane Library**

#1 MeSH descriptor: [Exercise] explode all trees

#2 MeSH descriptor: [Caloric Restriction] explode all trees

#3 MeSH descriptor: [Body Weight] explode all trees

#4 MeSH descriptor: [Body Mass Index] explode all trees

#5 MeSH descriptor: [Body Fat] explode all trees

#6 MeSH descriptor: [Body Fat Percentage] explode all trees

#7 MeSH descriptor: [Free Fat Mass] explode all trees

#8 #2 OR #3 OR #4 OR #5 OR #6 OR #7

#9 #1 AND #2 AND #8.

# Supplementary Figures and Tables

##
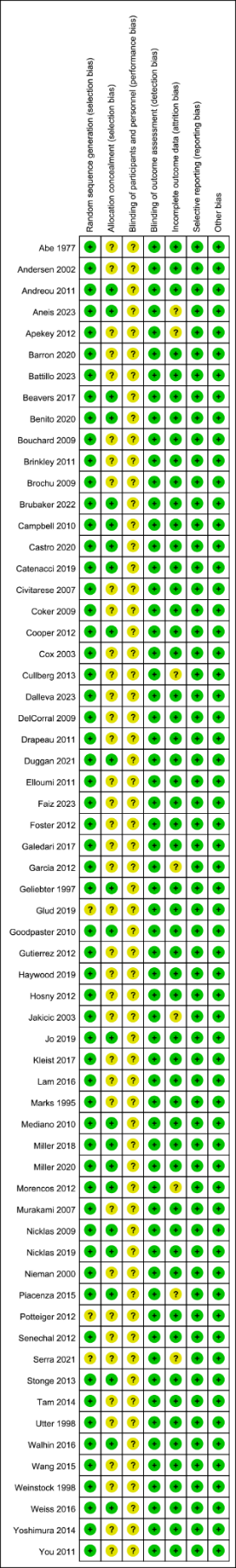
Supplementary Figures S1. Risk of bias summary

**Supplementary Figure S1**. Risk of bias summary

## Supplementary Figures S2. Risk of bias graph


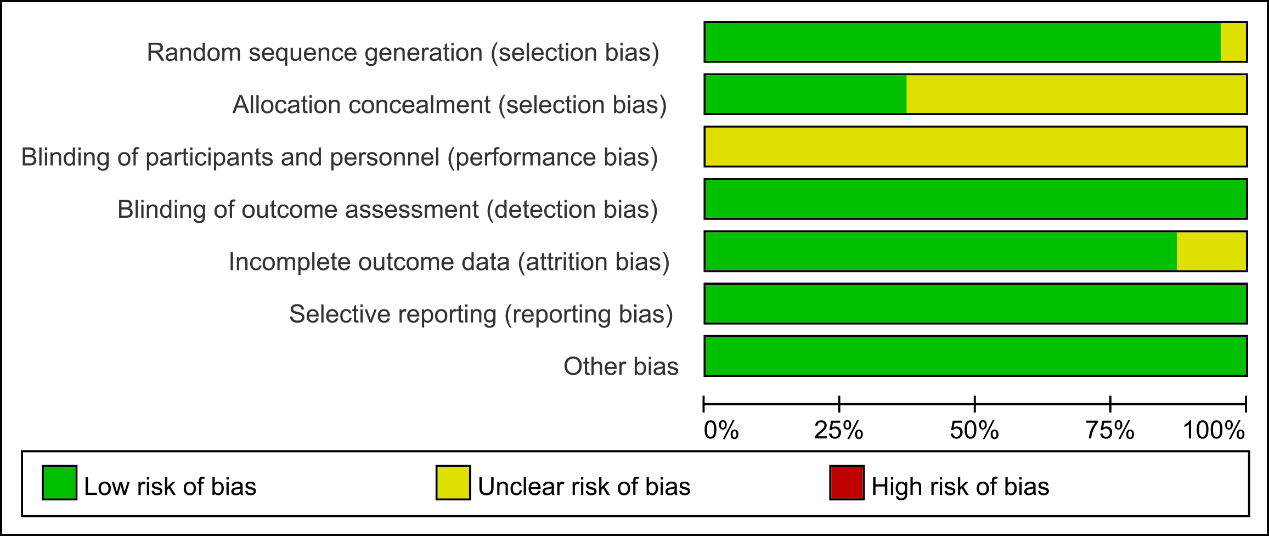


**Supplementary Figure S2**. Risk of bias graph

## Supplementary Table S1. Characteristic of included trials.

Table S1. Characteristic of included trials.

|  | Study | Sample Characteristics | Type of intervention | main indicators |
| --- | --- | --- | --- | --- |
| 1 | Abe 1977[1] | Sedentary young woman | 13 weeks, 1-2 times per week, moderate intensity aerobic exercise | BW, Fat%, Fat, FFM |
| 2 | Andersen 2002[2] | Overweight and obese adults | 12 weeks of high-intensity aerobic exercise 3 to 4 days per week | BW, Fat, FFM |
| 3 | Andreou 2011[3] | Overweight and/or obese women | 18 weeks of moderate-intensity aerobic exercise | BW, BMI, Fat%, FFM |
| 4 | Aneis 2023[4] | Obese premenopausal women | 12 weeks, mixed exercise, 60 minutes of exercise per day, three days per week, high-intensity mixed exercise | BMI |
| 5 | Apekey 2012[5] | Overweight/obese adults | 8 weeks, 30 minutes of low-intensity aerobic exercise twice a week | BW, BMI |
| 6 | Barron 2020[6] | Obese people | 16 weeks, 3-5 times per week, moderate intensity aerobic exercise | BW, BMI, Fat%, Fat, FFM |
| 7 | Battillo 2023[7] | Sedentary obese women | 2 weeks, 6 times per week, high intensity aerobic exercise | BW, BMI, Fat%, FFM |
| 8 | Beavers 2017[8] | Elderly | 6 months, 4 days per week, moderate intensity aerobic and resistance exercise | BW, Fat%, Fat, FFM |
| 9 | Benito 2020[9] | Overweight subjects | 22 weeks, 3 times per week, moderate intensity aerobic, resistance and mixed exercise | BW, BMI, Fat% |
| 10 | Bouchard 2009[10] | Obese postmenopausal women | 3 months of high-intensity resistance exercise 3 times per week on non-consecutive days | BW, BMI, Fat, FFM |
| 11 | Brinkley 2011[11] | Abdominal obesity in postmenopausal women | 20 weeks, 3 days a week, 50 minutes of moderate to vigorous intensity aerobic exercise | BW, BMI, Fat%, Fat, FFM |
| 12 | Brochu 2009[12] | Overweight postmenopausal women | 6 months, three times a week, high-intensity resistance exercise | BW, BMI |
| 13 | Brubaker 2022[13] | Overweight elderly | 20 weeks, 40 minutes 3 times per week, high-intensity aerobic and mixed exercise | BW, Fat%, Fat, FFM |
| 14 | Campbell 2010[14] | Obese people | 12 weeks, 30 minutes of moderate to high intensity aerobic exercise 5 times a week | BW, Fat, FFM |
| 15 | Castro 2020[15] | Overweight and obese men | 22 weeks, 3 times per week, moderate intensity aerobic, resistance and mixed exercise | BW, BMI, Fat%, FFM |
| 16 | Catenacci 2019[16] | Overweight or obese adults | 6 months, 300 minutes of high-intensity aerobic exercise per week | BW, Fat, FFM |
| 17 | Civitarese 2007[17] | Overweight young people | 6 months, 5 days a week, low-intensity aerobic exercise | BW, Fat%, Fat, FFM |
| 18 | Coker 2009[18] | Obese, elderly subjects | 12 weeks, 2500 kcal/week, moderate intensity aerobic exercise | BW, BMI, Fat% |
| 19 | Cooper 2012[19] | Severely obese participants | 6 months, 5 days a week, low-intensity aerobic exercise | BW, Fat, FFM |
| 20 | Cox 2003[20] | Overweight men | 16 weeks, 3 times a week, low-to-moderate intensity aerobic exercise | BW, Fat, FFM |
| 21 | Cullberg 2013[21] | Obese men and women | 12 weeks, 60-75 minutes of low-intensity aerobic exercise 3 times per week | BW, BMI |
| 22 | Dalleva 2023[22] | Obese male teenagers | 3 weeks, 30 minutes of moderate to high intensity aerobic exercise per day | BW, BMI, Fat%, Fat, FFM |
| 23 | DelCorral 2009[23] | Premenopausal women who are healthy and overweight (BMI 27-30) | 5 months, 3 times a week, high-intensity aerobic and resistance exercise | BW, BMI, Fat%, Fat, FFM |
| 24 | Drapeau 2011[24] | Overweight and obese postmenopausal women | 6 months, 3 times a week, moderate-intensity resistance exercise | BW, BMI, Fat%, Fat, FFM |
| 25 | Duggan 2021[25] | Overweight/obese, healthy, postmenopausal women | 12 months, 5 days a week, high intensity aerobic exercise | BW, BMI, Fat% |
| 26 | Elloumi 2011[26] | Male obese children | 2 months, 90 minutes of low-intensity aerobic exercise 4 times a week | BW, BMI, Fat, FFM |
| 27 | Faiz 2023[27] | Obese women | 13 days, 12 sessions of 60 minutes of high-intensity aerobic exercise | BW, BMI, Fat%, Fat, FFM |
| 28 | Foster 2012[28] | Overweight to obese postmenopausal sedentary women | 45 minutes of high-intensity aerobic exercise, 5 days a week, December | BW, BMI, Fat%, Fat, FFM |
| 29 | Galedari 2017[29] | Overweight and obese volunteers | 12 weeks, 3 days per week, moderate to high intensity aerobic and resistance exercise | BW, Fat%, FFM |
| 30 | Garcia 2012[30] | Obese women | 16 weeks, 2 times per week, high-intensity resistance exercise | BW, BMI |
| 31 | Geliebter 1997[31] | Moderately obese subjects | 8 weeks, 3 times a week, 60 minutes of moderate to high intensity aerobic and resistance exercise | BW, Fat, FFM |
| 32 | Glud 2019[32] | Overweight or obese but healthy individuals | 12 weeks, 3 times per week, high-intensity aerobic exercise | BW, BMI |
| 33 | Goodpaster 2010[33] | Adult participants with severe obesity (class II or III) without diabetes | 6 months, 5 days a week, 60 minutes of moderate intensity aerobic exercise | BW, BMI, Fat, FFM |
| 34 | Gutierrez 2012[34] | Obese people | 3 months, 30 minutes of moderate intensity aerobic exercise 3 times a week | , BMI, Fat% |
| 35 | Haywood 2019[35] | Obese elderly people | 12 weeks, 3 times per week, low-intensity aerobic exercise | BW, Fat, FFM |
| 36 | Hosny 2012[36] | Premenopausal obese women | 3 months, 40 minutes 3 times a week, high-intensity aerobic exercise | BW, BMI, FFM |
| 37 | Jakicic 2003[37] | Overweight women with a BMI of 27 to 40 | 12 months, 60 minutes of moderate to high intensity aerobic exercise per day | BW, BMI |
| 38 | Jo 2019[38] | Overweight and obese people | 12 weeks, 3 days per week, high-intensity resistance exercise | BW, BMI, Fat%, FFM |
| 39 | Kleist 2017[39] | Overweight and obese people | 12 weeks, 1 hour of moderate intensity aerobic exercise 3 times a week | BW, BMI, Fat, FFM |
| 40 | Lam 2016[40] | Overweight participants | 6 months to consume 12.5% of calories, low-intensity aerobic exercise | BW, BMI, Fat%, Fat, FFM |
| 41 | Marks 1995[41] | Overweight, inactive women | 20 weeks, 20-60 minutes of moderate to high intensity aerobic and resistance exercise 3-5 days per week | BW, Fat%, Fat, FFM |
| 42 | Mediano 2010[42] | Healthy women | 40 minutes of low-intensity aerobic exercise 3 times a week in December | BW, BMI, Fat% |
| 43 | Miller 2020[43] | Women with severe clinical obesity | 3 months, 2-3 times a week, high-intensity mixed exercise | BW, BMI, Fat, FFM |
| 44 | Miller 2018[44] | Overweight and obese premenstrual women | 16 weeks, 2-3 times per week, high-intensity resistance exercise | BW, Fat%, Fat, FFM |
| 45 | Morencos 2012[45] | Overweight adults | 22 weeks, 3 times per week, low-to-moderate intensity aerobic and resistance exercise | BW |
| 46 | Murakami 2007[46] | Obese adult volunteers | 12 weeks, 60 minutes of moderate-intensity aerobic exercise 3 days a week | BW, BMI, Fat |
| 47 | Nicklas 2019[47] | Older (65-79 years) obese men and women | 20 weeks, 30 minutes of moderate-intensity aerobic exercise 4 days a week | BW, Fat%, Fat, FFM |
| 48 | Nicklas 2009[48] | Overweight and obese women | 20 weeks, 55 minutes of moderate to high intensity aerobic exercise 3 times a week | BW, Fat%, Fat, FFM |
| 49 | Nieman 2000[49] | Obese women | 12 weeks, 45 minutes of vigorous-intensity aerobic exercise 5 times per week | BW, Fat% |
| 50 | Piacenza 2015[50] | Overweight people | 6 months, consuming 12.5% calories, low-intensity aerobic exercise | BW, BMI |
| 51 | Potteiger 2012[51] | Overweight men | 6 months, 45 minutes of high-intensity aerobic and resistance exercise, 4 days a week | BW, BMI, Fat%, Fat, FFM |
| 52 | Senechal 2012[52] | Postmenopausal obese women | 12 weeks, 3 times per week, low-intensity resistance exercise | BW, Fat%, Fat, FFM |
| 53 | Serra 2021[53] | Overweight and obese postmenopausal women | 6 months, 45 minutes of high-intensity aerobic exercise 3 days a week | BW, BMI |
| 54 | Stonge 2013[54] | Overweight and obese postmenopausal women | 6 months, 3 times a week, high-intensity resistance exercise | BW, Fat, FFM |
| 55 | Tam 2014[55] | Overweight men and women | 6 months 12.5% energy expenditure, low intensity aerobic exercise | BW, Fat |
| 56 | Utter 1998[56] | Obese women | 12 weeks, 45 minutes of vigorous-intensity aerobic exercise 5 times per week | BW, BMI, Fat%, Fat, FFM |
| 57 | Walhin 2016[57] | Overweight people | 3 weeks, 5 times a week, moderate to high intensity aerobic exercise | BW, Fat, FFM |
| 58 | Wang 2015[58] | Overweight or obese postmenopausal women | 5 months, 3 times a week, moderate intensity aerobic exercise | BW, BMI, Fat%, Fat, FFM |
| 59 | Weinstock 1998[59] | Obese women | 16 weeks, 3 times per week, moderate intensity aerobic and resistance exercise | BW, BMI |
| 60 | Weiss 2016[60] | Overweight, sedentary men and women | 14 weeks of low-intensity aerobic exercise with 10% energy expenditure per day | BW, BMI, Fat%, Fat, FFM |
| 61 | Yoshimura 2014[61] | Adults with visceral obesity | 12 weeks, 60 minutes of high-intensity aerobic exercise 5 times a week | BW, BMI, Fat%, Fat, FFM |
| 62 | You 2011[62] | Overweight or obese middle-aged women | 20 weeks, 55 minutes of moderate to high intensity aerobic exercise, 3 days per week | BW, Fat%, Fat |

# References

1. Abe, T.; Kawakami, Y.; Sugita, M.; Fukunaga, T. Relationship between Training Frequency and Subcutaneous and Visceral Fat in Women. *Medicine and science in sports and exercise* **1997**, *29*, 1549–1553, doi:10.1097/00005768-199712000-00001.

2. Andersen, R.E.; Franckowiak, S.C.; Bartlett, S.J.; Fontaine, K.R. Physiologic Changes after Diet Combined with Structured Aerobic Exercise or Lifestyle Activity. *Metabolism-Clinical and Experimental* **2002**, *51*, 1528–1533, doi:10.1053/meta.2002.36304.

3. Andreou, E.; Philippou, C.; Papandreou, D. Effects of an Intervention and Maintenance Weight Loss Diet with and without Exercise on Anthropometric Indices in Overweight and Obese Healthy Women. *Annals of nutrition & metabolism* **2011**, *59*, 187‐192, doi:10.1159/000334755.

4. Aneis, Y.M.; El Refaye, G.E.; Taha, M.M.; Aldhahi, M.I.; Elsisi, H.F. Concurrent Aerobic and Strength Training with Caloric Restriction Reduces Insulin Resistance in Obese Premenopausal Women: A Randomized Controlled Trial. *Medicina (Kaunas, Lithuania)* **2023**, *59*, doi:10.3390/medicina59071193.

5. Apekey, T.A.; Morris, A.E.J.; Fagbemi, S.; Griffiths, G.J. Benefits of Moderate-Intensity Exercise during a Calorie-Restricted Low-Fat Diet. *Health Education Journal* **2012**, *71*, 154–164, doi:10.1177/0017896911398235.

6. Barron-Cabrera, E.; Gonzalez-Becerra, K.; Rosales-Chavez, G.; Mora-Jimenez, A.; Hernandez-Canaveral, I.; Martinez-Lopez, E. Low-Grade Chronic Inflammation Is Attenuated by Exercise Training in Obese Adults through down-Regulation of *ASC* Gene in Peripheral Blood: A Pilot Study. *Genes and Nutrition* **2020**, *15*, doi:10.1186/s12263-020-00674-0.

7. Battillo, D.J.; Malin, S.K. Impact of Caloric Restriction and Exercise on Trimethylamine N-Oxide Metabolism in Women with Obesity. *Nutrients* **2023**, *15*, doi:10.3390/nu15061455.

8. Beavers, K.M.; Ambrosius, W.T.; Rejeski, W.J.; Burdette, J.H.; Walkup, M.P.; Sheedy, J.L.; Nesbit, B.A.; Gaukstern, J.E.; Nicklas, B.J.; Marsh, A.P. Effect of Exercise Type During Intentional Weight Loss on Body Composition in Older Adults with Obesity. *Obesity* **2017**, *25*, 1823–1829, doi:10.1002/oby.21977.

9. Benito, P.J.; Lopez-Plaza, B.; Bermejo, L.M.; Peinado, A.B.; Cupeiro, R.; Butragueno, J.; Rojo-Tirado, M.A.; Gonzalez-Lamuno, D.; Gomez-Candela, C.; Pronaf Study Grp Strength plus Endurance Training and Individualized Diet Reduce Fat Mass in Overweight Subjects: A Randomized Clinical Trial. *International Journal of Environmental Research and Public Health* **2020**, *17*, doi:10.3390/ijerph17072596.

10. Bouchard, D.R.; Soucy, L.; Sénéchal, M.; Dionne, I.J.; Brochu, M. Impact of Resistance Training with or without Caloric Restriction on Physical Capacity in Obese Older Women. *Menopause (New York, N.Y.)* **2009**, *16*, 66‐72, doi:10.1097/gme.0b013e31817dacf7.

11. Brinkley, T.E.; Wang, X.; Kume, N.; Mitsuoka, H.; Nicklas, B.J. Caloric Restriction, Aerobic Exercise Training and Soluble Lectin-like Oxidized LDL Receptor-1 Levels in Overweight and Obese Post-Menopausal Women. *International journal of obesity (2005)* **2011**, *35*, 793‐799, doi:10.1038/ijo.2010.199.

12. Brochu, M.; Malita, M.F.; Messier, V.; Doucet, E.; Strychar, I.; Lavoie, J.M.; Prud’homme, D.; Rabasa-Lhoret, R. Resistance Training Does Not Contribute to Improving the Metabolic Profile after a 6-Month Weight Loss Program in Overweight and Obese Postmenopausal Women. *Journal of clinical endocrinology and metabolism* **2009**, *94*, 3226‐3233, doi:10.1210/jc.2008-2706.

13. Brubaker, P.H.; Nicklas, B.; Houston, D.; Hundley, W.G.; Chen, H.; Molina, A.; Lyles, M.; Nelson, M.; Upadhya, B.; Newland, R.; et al. A Randomized, Controlled Trial of Resistance Training Added to Caloric Restriction Plus Aerobic Exercise Training in Obese Heart Failure With Preserved Ejection Fraction. *Circulation* **2022**, *146*, doi:10.1161/circ.146.suppl_1.11974.

14. Campbell, L.; Wallman, K.; Green, D. The Effects of Intermittent Exercise on Physiological Outcomes in an Obese Population: Continuous versus Interval Walking. *Journal of Sports Science and Medicine* **2010**, *9*, 24–30.

15. Castro, E.A.; Carraça, E.V.; Cupeiro, R.; López-Plaza, B.; Teixeira, P.J.; González-Lamuño, D.; Peinado, A.B. The Effects of the Type of Exercise and Physical Activity on Eating Behavior and Body Composition in Overweight and Obese Subjects. *Nutrients* **2020**, *12*, doi:10.3390/nu12020557.

16. Catenacci, V.A.; Ostendorf, D.M.; Pan, Z.; Bing, K.; Wayland, L.T.; Seyoum, E.; Stauffer, B.L.; Phelan, S.; Creasy, S.A.; Caldwell, A.E.; et al. The Impact of Timing of Exercise Initiation on Weight Loss: An 18-Month Randomized Clinical Trial. *Obesity* **2019**, *27*, 1828–1838, doi:10.1002/oby.22624.

17. Civitarese, A.E.; Carling, S.; Heilbronn, L.K.; Hulver, M.H.; Ukropcova, B.; Deutsch, W.A.; Smith, S.R.; Ravussin, E. Calorie Restriction Increases Muscle Mitochondrial Biogenesis in Healthy Humans. *PLoS Med* **2007**, *4*, e76, doi:10.1371/journal.pmed.0040076.

18. Coker, R.H.; Williams, R.H.; Yeo, S.E.; Kortebein, P.M.; Bodenner, D.L.; Kern, P.A.; Evans, W.J. The Impact of Exercise Training Compared to Caloric Restriction on Hepatic and Peripheral Insulin Resistance in Obesity. *Journal of clinical endocrinology and metabolism* **2009**, *94*, 4258‐4266, doi:10.1210/jc.2008-2033.

19. Cooper, J.N.; Columbus, M.L.; Shields, K.J.; Asubonteng, J.; Meyer, M.L.; Sutton-Tyrrell, K.; Goodpaster, B.H.; DeLany, J.P.; Jakicic, J.M.; Barinas-Mitchell, E. Effects of an Intensive Behavioral Weight Loss Intervention Consisting of Caloric Restriction with or without Physical Activity on Common Carotid Artery Remodeling in Severely Obese Adults. *Metabolism: clinical and experimental* **2012**, *61*, 1589‐1597, doi:10.1016/j.metabol.2012.04.012.

20. Cox, K.L.; Burke, V.; Morton, A.R.; Beilin, L.J.; Puddey, I.B. The Independent and Combined Effects of 16 Weeks of Vigorous Exercise and Energy Restriction on Body Mass and Composition in Free-Living Overweight Men--a Randomized Controlled Trial. *Metabolism: clinical and experimental* **2003**, *52*, 107‐115, doi:10.1053/meta.2003.50017.

21. Cullberg, K.B.; Christiansen, T.; Paulsen, S.K.; Bruun, J.M.; Pedersen, S.B.; Richelsen, B. Effect of Weight Loss and Exercise on Angiogenic Factors in the Circulation and in Adipose Tissue in Obese Subjects. *Obesity* **2013**, *21*, 454–460, doi:10.1002/oby.20060.

22. D’Alleva, M.; Lazzer, S.; Tringali, G.; De Micheli, R.; Bondesan, A.; Abbruzzese, L.; Sartorio, A. Effects of Combined Training or Moderate Intensity Continuous Training during a 3-Week Multidisciplinary Body Weight Reduction Program on Cardiorespiratory Fitness, Body Composition, and Substrate Oxidation Rate in Adolescents with Obesity. *Scientific reports* **2023**, *13*, doi:10.1038/s41598-023-44953-3.

23. Del Corral, P.; Chandler-Laney, P.C.; Casazza, K.; Gower, B.A.; Hunter, G.R. Effect of Dietary Adherence with or without Exercise on Weight Loss: A Mechanistic Approach to a Global Problem. *Journal of clinical endocrinology and metabolism* **2009**, *94*, 1602‐1607, doi:10.1210/jc.2008-1057.

24. Drapeau, S.; Doucet, E.; Rabasa-Lhoret, R.; Brochu, M.; Prud’homme, D.; Imbeault, P. Improvement in Insulin Sensitivity by Weight Loss Does Not Affect Hyperinsulinemia-Mediated Reduction in Total and High Molecular Weight Adiponectin: A MONET Study. *Physiologie appliquee, nutrition et metabolisme [Applied physiology, nutrition, and metabolism]* **2011**, *36*, 191‐200, doi:10.1139/h10-106.

25. Duggan, C.; De Dieu Tapsoba, J.; Shivappa, N.; Harris, H.R.; Hébert, J.R.; Wang, C.Y.; McTiernan, A. Changes in Dietary Inflammatory Index Patterns with Weight Loss in Women: A Randomized Controlled Trial. *Cancer Prevention Research* **2021**, *14*, 85–94, doi:10.1158/1940-6207.CAPR-20-0181.

26. Elloumi, M.; Makni, E.; Ounis, O.B.; Moalla, W.; Zbidi, A.; Zaoueli, M.; Lac, G.; Tabka, Z. Six-Minute Walking Test and the Assessment of Cardiorespiratory Responses during Weight-Loss Programmes in Obese Children. *Physiotherapy research international* **2011**, *16*, 32‐42, doi:10.1002/pri.470.

27. Faiz, H.; Malin, S.K. A Low-Calorie Diet Raises β-Aminoisobutyric Acid in Relation to Glucose Regulation and Leptin Independent of Exercise in Women with Obesity. *Frontiers in Physiology* **2023**, *14*, doi:10.3389/fphys.2023.1210567.

28. Foster-Schubert, K.E.; Alfano, C.M.; Duggan, C.R.; Xiao, L.; Campbell, K.L.; Kong, A.; Bain, C.E.; Wang, C.Y.; Blackburn, G.L.; McTiernan, A. Effect of Diet and Exercise, Alone or Combined, on Weight and Body Composition in Overweight-to-Obese Postmenopausal Women. *Obesity (Silver Spring, Md.)* **2012**, *20*, 1628‐1638, doi:10.1038/oby.2011.76.

29. Galedari, M.; Azarbayjani, M.A.; Peeri, M. Effects of Type of Exercise along with Caloric Restriction on Plasma Apelin 36 and HOMA-IR in Overweight Men. *Science and Sports* **2017**, *32*, e137–e145, doi:10.1016/j.scispo.2016.12.002.

30. Garcia-Unciti, M.; Izquierdo, M.; Idoate, F.; Gorostiaga, E.; Grijalba, A.; Ortega-Delgado, F.; Martinez-Labari, C.; Moreno-Navarrete, J.M.; Forga, L.; Manuel Fernandez-Real, J.; et al. Weight-Loss Diet Alone or Combined with Progressive Resistance Training Induces Changes in Association between the Cardiometabolic Risk Profile and Abdominal Fat Depots. *Annals of Nutrition and Metabolism* **2012**, *61*, 296–304, doi:10.1159/000342467.

31. Geliebter, A.; Maher, M.M.; Gerace, L.; Gutin, B.; Heymsfield, S.B.; Hashim, S.A. Effects of Strength or Aerobic Training on Body Composition, Resting Metabolic Rate, and Peak Oxygen Consumption in Obese Dieting Subjects. *American journal of clinical nutrition* **1997**, *66*, 557–563, doi:10.1093/ajcn/66.3.557.

32. Glud, M.; Christiansen, T.; Larsen, L.H.; Richelsen, B.; Bruun, J.M. Changes in Circulating BDNF in Relation to Sex, Diet, and Exercise: A 12-Week Randomized Controlled Study in Overweight and Obese Participants. *Journal of obesity* **2019**, *2019*, doi:10.1155/2019/4537274.

33. Goodpaster, B.H.; DeLany, J.P.; Otto, A.D.; Kuller, L.; Vockley, J.; South-Paul, J.E.; Thomas, S.B.; Brown, J.; McTigue, K.; Hames, K.C.; et al. Effects of Diet and Physical Activity Interventions on Weight Loss and Cardiometabolic Risk Factors in Severely Obese Adults A Randomized Trial. *Jama-Journal of the American Medical Association* **2010**, *304*, 1795–1802, doi:10.1001/jama.2010.1505.

34. Gutierrez-Lopez, L.; Garcia-Sanchez, J.R.; Rincon-Viquez Mde, J.; Lara-Padilla, E.; Sierra-Vargas, M.P.; Olivares-Corichi, I.M. Hypocaloric Diet and Regular Moderate Aerobic Exercise Is an Effective Strategy to Reduce Anthropometric Parameters and Oxidative Stress in Obese Patients. *Obesity facts* **2012**, *5*, 12‐22, doi:10.1159/000336526.

35. Haywood, C.J.; Prendergast, L.A.; Lim, R.; Lappas, M.; Lim, W.K.; Proietto, J. Obesity in Older Adults: Effect of Degree of Weight Loss on Cardiovascular Markers and Medications. *Clinical Obesity* **2019**, *9*, doi:10.1111/cob.12316.

36. Hosny, I.A.; Elghawabi, H.S.; Younan, W.B.; Sabbour, A.A.; Gobrial, M.A. Beneficial Impact of Aerobic Exercises on Bone Mineral Density in Obese Premenopausal Women under Caloric Restriction. *Skeletal radiology* **2012**, *41*, 423‐427, doi:10.1007/s00256-011-1196-1.

37. Jakicic, J.M.; Marcus, B.H.; Gallagher, K.I.; Napolitano, M.; Lang, W. Effect of Exercise Duration and Intensity on Weight Loss in Overweight, Sedentary Women: A Randomized Trial. *JAMA* **2003**, *290*, 1323–1330, doi:10.1001/jama.290.10.1323.

38. Jo, E.; Worts, P.R.; Elam, M.L.; Brown, A.F.; Khamoui, A.V.; Kim, D.-H.; Yeh, M.-C.; Ormsbee, M.J.; Prado, C.M.; Cain, A.; et al. Resistance Training during a 12-Week Protein Supplemented VLCD Treatment Enhances Weight-Loss Outcomes in Obese Patients. *Clinical Nutrition* **2019**, *38*, 372–382, doi:10.1016/j.clnu.2017.12.015.

39. Kleist, B.; Wahrburg, U.; Stehle, P.; Schomaker, R.; Greiwing, A.; Stoffel-Wagner, B.; Egert, S. Moderate Walking Enhances the Effects of an Energy-Restricted Diet on Fat Mass Loss and Serum Insulin in Overweight and Obese Adults in a 12-Week Randomized Controlled Trial. *Journal of Nutrition* **2017**, *147*, 1875–1884, doi:10.3945/jn.117.251744.

40. Lam, Y.Y.; Ghosh, S.; Civitarese, A.E.; Ravussin, E. Six-Month Calorie Restriction in Overweight Individuals Elicits Transcriptomic Response in Subcutaneous Adipose Tissue That Is Distinct From Effects of Energy Deficit. *Journals of gerontology. Series A, Biological sciences and medical sciences* **2016**, *71*, 1258‐1265, doi:10.1093/gerona/glv194.

41. Marks, B.L.; Ward, A.; Morris, D.H.; Castellani, J.; Rippe, J.M. Fat-Free Mass Is Maintained in Women Following a Moderate Diet and Exercise Program. *Medicine and science in sports and exercise* **1995**, *27*, 1243–1251.

42. Mediano, M.F.; Barbosa, J.S.; Moura, A.S.; Willett, W.C.; Sichieri, R. A Randomized Clinical Trial of Home-Based Exercise Combined with a Slight Caloric Restriction on Obesity Prevention among Women. *Preventive medicine* **2010**, *51*, 247‐252, doi:10.1016/j.ypmed.2010.07.012.

43. Miller, C.T.; Fraser, S.F.; Selig, S.E.; Rice, T.; Grima, M.; van den Hoek, D.J.; Ika Sari, C.; Lambert, G.W.; Dixon, J.B. Fitness, Strength and Body Composition during Weight Loss in Women with Clinically Severe Obesity: A Randomised Clinical Trial. *Obesity facts* **2020**, *13*, 307–321, doi:10.1159/000506643.

44. Miller, T.; Mull, S.; Aragon, A.A.; Krieger, J.; Schoenfeld, B.J. Resistance Training Combined With Diet Decreases Body Fat While Preserving Lean Mass Independent of Resting Metabolic Rate: A Randomized Trial. *International journal of sport nutrition and exercise metabolism* **2018**, *28*, 46–54, doi:10.1123/ijsnem.2017-0221.

45. Morencos, E.; Romero, B.; Peinado, A.B.; González-Gross, M.; Fernández, C.; Gómez-Candela, C.; Benito, P.J. Effects of Dietary Restriction Combined with Different Exercise Programs or Physical Activity Recommendations on Blood Lipids in Overweight Adults. *Nutricion hospitalaria* **2012**, *27*, 1916‐1927, doi:10.3305/nh.2012.27.6.6057.

46. Murakami, T.; Horigome, H.; Tanaka, K.; Nakata, Y.; Katayama, Y.; Matsui, A. Effects of Diet with or without Exercise on Leptin and Anticoagulation Proteins Levels in Obesity. *Blood Coagulation and Fibrinolysis* **2007**, *18*, 389–394, doi:10.1097/01.mbc.0000278929.87251.5d.

47. Nicklas, B.J.; Brinkley, T.E.; Houston, D.K.; Lyles, M.F.; Hugenschmidt, C.E.; Beavers, K.M.; Leng, X. Effects of Caloric Restriction on Cardiorespiratory Fitness, Fatigue, and Disability Responses to Aerobic Exercise in Older Adults With Obesity: A Randomized Controlled Trial. *J Gerontol A Biol Sci Med Sci* **2019**, *74*, 1084–1090, doi:10.1093/gerona/gly159.

48. Nicklas, B.J.; Wang, X.; You, T.; Lyles, M.F.; Demons, J.; Easter, L.; Berry, M.J.; Lenchik, L.; Carr, J.J. Effect of Exercise Intensity on Abdominal Fat Loss during Calorie Restriction in Overweight and Obese Postmenopausal Women: A Randomized, Controlled Trial. *Am J Clin Nutr* **2009**, *89*, 1043–1052, doi:10.3945/ajcn.2008.26938.

49. Nieman, D.C.; Custer, W.F.; Butterworth, D.E.; Utter, A.C.; Henson, D.A. Psychological Response to Exercise Training and/or Energy Restriction in Obese Women. *Journal of Psychosomatic Research* **2000**, *48*, 23–29, doi:10.1016/S0022-3999(99)00066-5.

50. Piacenza, F.; Malavolta, M.; Basso, A.; Costarelli, L.; Giacconi, R.; Ravussin, E.; Redman, L.M.; Mocchegiani, E. Effect of 6-Month Caloric Restriction on Cu Bound to Ceruloplasmin in Adult Overweight Subjects. *Journal of nutritional biochemistry* **2015**, *26*, 876‐882, doi:10.1016/j.jnutbio.2015.03.012.

51. Potteiger, J.A.; Claytor, R.P.; Hulver, M.W.; Hughes, M.R.; Carper, M.J.; Richmond, S.; Thyfault, J.P. Resistance Exercise and Aerobic Exercise When Paired with Dietary Energy Restriction Both Reduce the Clinical Components of Metabolic Syndrome in Previously Physically Inactive Males. *European journal of applied physiology* **2012**, *112*, 2035‐2044, doi:10.1007/s00421-011-2174-y.

52. Senechal, M.; Bouchard, D.R.; Dionne, I.J.; Brochu, M. The Effects of Lifestyle Interventions in Dynapenic-Obese Postmenopausal Women. *Menopause (New York, N.Y.)* **2012**, *19*, 1015‐1021, doi:10.1097/gme.0b013e318248f50f.

53. Serra, M.C.; Ryan, A.S. Bone Mineral Density Changes during Weight Regain Following Weight Loss with and without Exercise. *Nutrients* **2021**, *13*, doi:10.3390/nu13082848.

54. St-Onge, M.; Rabasa-Lhoret, R.; Strychar, I.; Faraj, M.; Doucet, É.; Lavoie, J.M. Impact of Energy Restriction with or without Resistance Training on Energy Metabolism in Overweight and Obese Postmenopausal Women: A Montreal Ottawa New Emerging Team Group Study. *Menopause (New York, N.Y.)* **2013**, *20*, 194‐201, doi:10.1097/gme.0b013e318261f22a.

55. Tam, C.S.; Frost, E.A.; Xie, W.; Rood, J.; Ravussin, E.; Redman, L.M. No Effect of Caloric Restriction on Salivary Cortisol Levels in Overweight Men and Women. *Metabolism: clinical and experimental* **2014**, *63*, 194‐198, doi:10.1016/j.metabol.2013.10.007.

56. Utter, A.C.; Nieman, D.C.; Shannonhouse, E.M.; Butterworth, D.E.; Nieman, C.N. Influence of Diet and/or Exercise on Body Composition and Cardiorespiratory Fitness in Obese Women. *International Journal of Sport Nutrition* **1998**, *8*, 213–222, doi:10.1123/ijsn.8.3.213.

57. Walhin, J.P.; Dixon, N.C.; Betts, J.A.; Thompson, D. The Impact of Exercise Intensity on Whole Body and Adipose Tissue Metabolism during Energy Restriction in Sedentary Overweight Men and Postmenopausal Women. *Physiological reports* **2016**, *4*, doi:10.14814/phy2.13026.

58. Wang, X.; You, T.; Murphy, K.; Lyles, M.F.; Nicklas, B.J. Addition of Exercise Increases Plasma Adiponectin and Release from Adipose Tissue. *Medicine and science in sports and exercise* **2015**, *47*, 2450‐2455, doi:10.1249/MSS.0000000000000670.

59. Weinstock, R.S.; Dai, H.L.; Wadden, T.A. Diet and Exercise in the Treatment of Obesity -: *Effects of 3 Interventions on Insulin Resistance*. *Archives of internal medicine* **1998**, *158*, 2477–2483, doi:10.1001/archinte.158.22.2477.

60. Weiss, E.P.; Albert, S.G.; Reeds, D.N.; Kress, K.S.; McDaniel, J.L.; Klein, S.; Villareal, D.T. Effects of Matched Weight Loss from Calorie Restriction, Exercise, or Both on Cardiovascular Disease Risk Factors: A Randomized Intervention Trial. *American journal of clinical nutrition* **2016**, *104*, 576‐586, doi:10.3945/ajcn.116.131391.

61. Yoshimura, E.; Kumahara, H.; Tobina, T.; Matsuda, T.; Ayabe, M.; Kiyonaga, A.; Anzai, K.; Higaki, Y.; Tanaka, H. Lifestyle Intervention Involving Calorie Restriction with or without Aerobic Exercise Training Improves Liver Fat in Adults with Visceral Adiposity. *Journal of obesity* **2014**, *2014*, 197216, doi:10.1155/2014/197216.

62. You, T.; Disanzo, B.L.; Wang, X.; Yang, R.; Gong, D. Adipose Tissue Endocannabinoid System Gene Expression: Depot Differences and Effects of Diet and Exercise. *Lipids in Health and Disease* **2011**, *10*, doi:10.1186/1476-511X-10-194.
